# Bronchial foreign body caused by a grasshopper in a dog

**DOI:** 10.1007/s11259-025-10687-y

**Published:** 2025-02-24

**Authors:** Giulia Maggi, Elena Rosi, Simone Cupido, Salvatore Palma, Maria Chiara Marchesi

**Affiliations:** Department of Veterinary Medicine, University of Perugia, Via San Costanzo 4, Perugia, 06126 Italy

**Keywords:** Bronchial foreign body, Endoscopy, Dog, Grasshopper, Cough

## Abstract

An 11-year-old dog living in a rural area of Italy was evaluated for a persistent cough. Diagnostic imaging findings, combined with the clinical history and physical examination, revealed focal pneumonia, raising suspicion of a vegetal foreign body. Bronchoscopy identified a foreign body (a grasshopper) partially obstructing the left caudal bronchus, and its removal was successfully performed. Insects should be considered potential bronchial foreign bodies in the differential diagnosis.

## Background

In companion animals, bronchial foreign bodies (FBs) are predominantly vegetal in origin (Cerquetella et al. 2013; Flageollet et al. 2023). Additionally, cases involving stones, bullets, nails, bone fragments, and aspirated teeth have been documented (Pacchiana et al. 2001; Volta et al. 2007). According to the literature, FBs are most commonly localized in the right bronchi, particularly in the segmental branches of the right main bronchus (Cerquetella et al. 2013). This prevalence is attributed to the anatomical configuration of the bronchial bifurcation, as the right main bronchus is almost a direct continuation of the trachea. In rare cases, FBs are found in the left bronchi (Cerquetella et al. 2013). Cough is one of the most common clinical signs associated with bronchial FBs. Occasionally, dyspnea may also occur, especially when bacterial pneumonia develops as a consequence of their presence. Radiographic and ultrasonographic findings may be absent or reveal signs of focal pneumonia. Endoscopic examination of the bronchi allows for a definitive diagnosis of bronchial FBs and can also be useful for treatment, which involves the removal of the FBs (De Lorenzi 2012).

## Case presentation

An 11-year-old neutered male hound dog, weighing 25 kg and living indoors with access to the outdoors in a rural area, presented to the Veterinary Teaching Hospital (VTH) of the University of Perugia in July due to a persistent cough. On clinical examination, the dog appeared alert, with normal mucous membranes, a capillary refill time (CRT) of < 2 sec, tachypnea (60 breaths/min), a heart rate of 90 bpm, and a rectal temperature of 38.5 °C. Thoracic auscultation revealed a reinforced vesicular murmur and a slight decrease in breath sounds in the mid-caudal region of the left hemithorax. Complete Blood Count (CBC) and biochemical examination were performed, revealing a slight elevation in White Blood Cell Count (WBC = 20.4 10^3^/ µL), primarily due to an increased neutrophil percentage (84.4%).

**Fig. 1:**
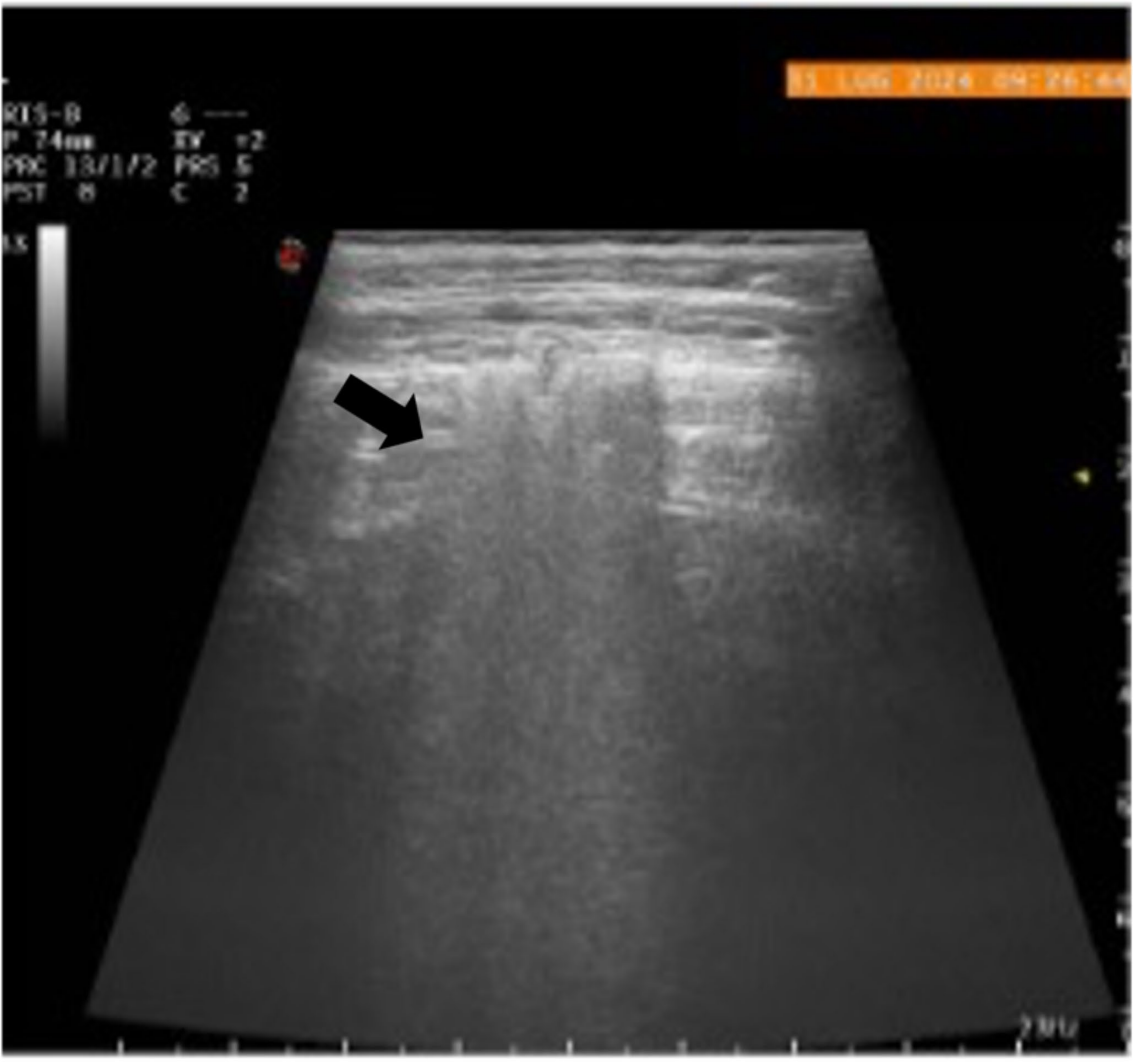
Ultrasound examination revealed a subpleural hypoechoic area surrounded by an interstitial pattern, consistent with pneumonia

The ultrasonographic evaluation of the left hemithorax revealed a small subpleural hypoechoic area surrounded by an interstitial pattern, consistent with a focal area of pneumonia (Fig. 1). The right hemithorax showed no abnormalities.

Lateral-lateral (LL) and ventro-dorsal (VD) radiographs of the neck and thorax were performed, revealing a radiopacity with a mixed bronchial and interstitial pattern in the left caudal lobe.

The combination of ultrasonographic and radiographic findings, along with the clinical history, raised suspicion of a vegetal FB, prompting an endoscopic examination.

The dog underwent a trachea-bronchoscopic examination under general anesthesia using a 6 mm x 100 cm flexible videoendoscope (Pentax EG-1840, Pentax, Tokyo, Japan). Premedication included dexmedetomidine (2 µg/kg) and butorphanol (0.2 mg/kg) administered intramuscularly. Anesthesia was induced with propofol (4 mg/kg) administered intravenously and maintained through Total Intravenous Anesthesia (TIVA) with propofol, starting at a dose of 0.4 mg/kg/min and gradually reduced to 0.2 mg/kg/min.

**Fig. 2:**
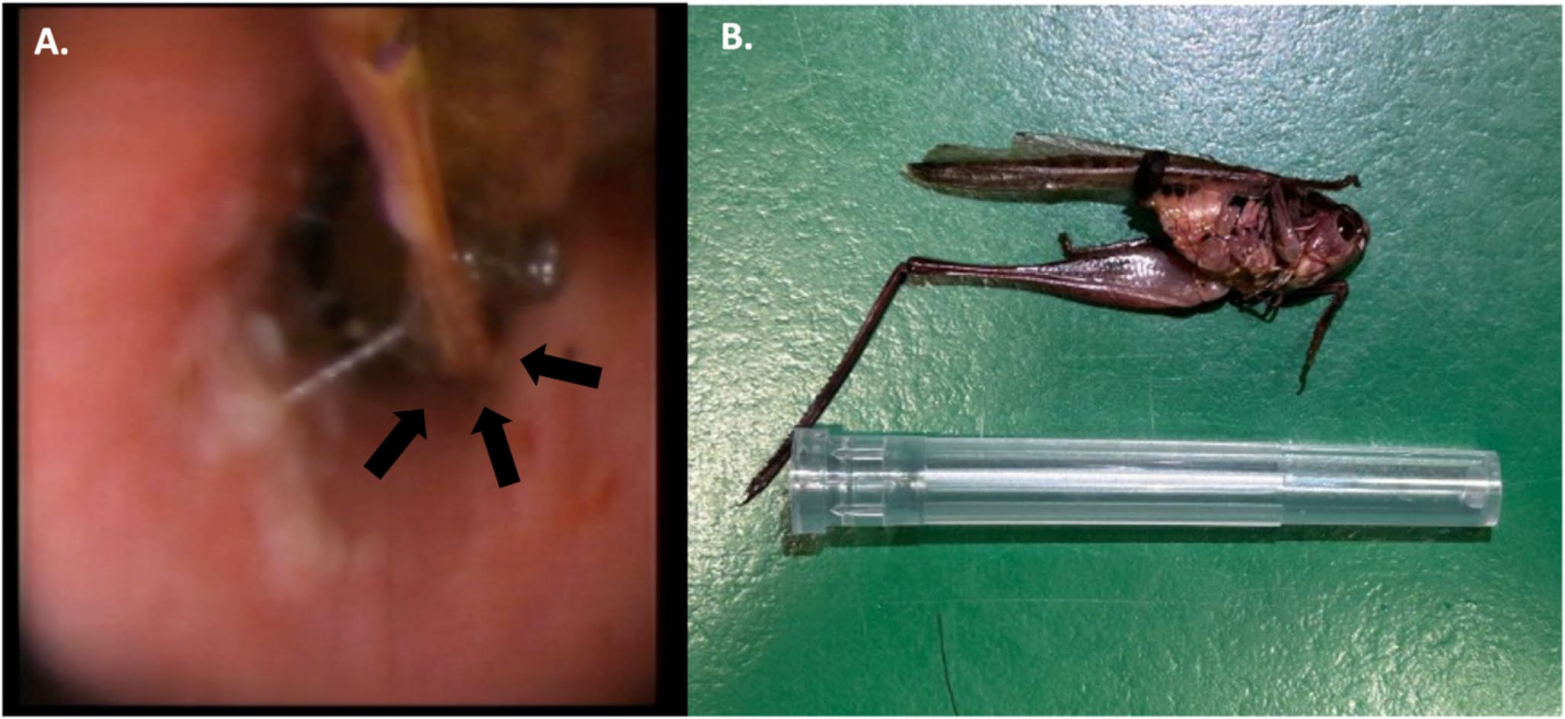
Bronchial foreign body. (**A**) Endoscopic examination revealed a grasshopper causing subocclusion of LB2; (**B**) The grasshopper after extraction

Bronchoscopy revealed a FB (a 1.7 cm grasshopper, *Decticus verrucivorus*) partially obstructing the left caudal bronchus (LB2, according to the Amis and McKiernan classification (1986)), along with mucopurulent exudate (Fig. 2). The FB was successfully removed with rat-tooth forcep inserted through the endoscope’s working channel.

The dog was discharged with a 15-day course of marbofloxacin (2 mg/kg once daily), leading to a rapid remission of symptoms. A follow-up bronchoscopy performed two weeks later confirmed the absence of additional FBs or exudate.

## Discussion and conclusions

This case report describes a grasshopper suboccluding a left caudal bronchus, causing coughing as a clinical sign in a dog living in a rural area of Italy. To the best of our knowledge, this is the first report of an insect as a bronchial FB. Diagnostic imaging (ultrasonography and radiography) revealed findings consistent with focal pneumonia, which, combined with the clinical history and clinical examination, raised suspicion of a bronchial vegetal FB. Endoscopic examination of the lower airways revealed the presence of an insect as the FB and enabled its extraction. We hypothesize an etiopathogenesis similar to that of vegetal FBs, in which penetration into the bronchi occurs through the glottis. In active dogs, the glottis remains persistently open to enhance oxygenation, facilitating the penetration of FBs. This anatomical configuration explains the common localization of bronchial FBs in the right main bronchus, which is nearly a direct continuation of the trachea (De Lorenzi 2012). For these reasons, and due to the increased likelihood of contact with grass awns when dogs are working or engaging in outdoor activities in rural areas, bronchial FBs are frequently observed in hunting dogs. The aspiration of vegetal FBs is even considered an occupational disease in these animals (Marchesi et al. 2019). To support our hypothesis, the animal described in our clinical case was a hunting dog. The grasshopper was localized and retrieved from the left caudal bronchus, a less commonly reported location in the literature on bronchial FBs (Cerquetella et al. 2013). The most common clinical sign in animals with bronchial FBs is coughing, which results from the persistent presence of the FB, as well as from bronchoconstriction and mucus hypersecretion, representing the local bronchial response (Marchesi et al. 2019). Cough secondary to vegetal FBs typically has an acute onset and a seasonal pattern. This respiratory condition most commonly occurs in spring and summer; however, cases can also be diagnosed in other seasons, often originating during the warmer months but remaining undiagnosed. In these cases, when the anamnesis is thoroughly investigated, the onset of clinical signs is usually traced back to the spring or summer (Marchesi et al. 2019; De Lorenzi 2012). In our case report, the clinical signs had an acute onset in summer (July), which could be associated with the presence of adult grasshoppers during warmer seasons (Simpson and Sword 2009). The similarity in the clinical onset and presentation of grass awn and insect bronchial aspiration underscores the importance of considering this possibility in the differential diagnosis of bronchial vegetal FBs. Dog fully recovered from its symptoms following the procedure and a course of broad-spectrum antibiotic therapy. Bronchoscopy remains a valuable tool for both diagnosing and removing bronchial FBs. Although aspiration of insects in dogs frequenting rural areas is uncommon, it should be considered a potential cause alongside the more typical vegetal FBs.

## Data Availability

No datasets were generated or analysed during the current study.
